# Transgenerational effect of prenatal stress on behavior and lipid peroxidation in brain structures of female rats during the estral cycle

**DOI:** 10.18699/vjgb-24-44

**Published:** 2024-07

**Authors:** A.V. Vyushina, A.V. Pritvorova, S.G. Pivina, N.E. Ordyan

**Affiliations:** Pavlov Institute of Physiology, Russian Academy of Sciences, St. Petersburg, Russia; Pavlov Institute of Physiology, Russian Academy of Sciences, St. Petersburg, Russia; Pavlov Institute of Physiology, Russian Academy of Sciences, St. Petersburg, Russia; Pavlov Institute of Physiology, Russian Academy of Sciences, St. Petersburg, Russia

**Keywords:** prenatal stress, F2 generation, behavior, lipid peroxidation, estrus, diestrus, пренатальный стресс, поколение F2, поведение, перекисное окисление липидов, эструс, диэструс

## Abstract

The effect of stress in pregnant female Wistar rats on the behavior and lipid peroxidation (LP) in the neocortex, hippocampus and hypothalamus in the female F2 generation during the ovarian cycle was investigated. We subjected pregnant females to daily 1-hour immobilization stress from the 15th to the 19th days of pregnancy. Further, family groups were formed from prenatally stressed and control male and female rats of the F1 generation: group 1, the control female and male; group 2, the control female and the prenatally stressed male; group 3, the prenatally stressed female and the control male; group 4, the prenatally stressed female and male. The females of the F2 generation born from these couples were selected into four experimental groups in accordance with the family group. At the age of 3 months, behavior of rats was studied in the “open field” test in two stages of the ovarian cycle – estrus and diestrus. After 7–10 days, the rats were decapitated and the neocortex, hypothalamus and hippocampus were selected to determine the level of diene and triene conjugates, Schiff bases and the degree of lipid oxidation (Klein index). In F2 females with one prenatally stressed parent, there was no interstage difference in locomotor-exploratory activity and anxiety. If both F1 parents were prenatally stressed, female F2 rats retained interstage differences similar to the control pattern, while their locomotor-exploratory activity and time spent in the center of an “open field” decreased in absolute values. In the neocortex of F2 females in groups with prenatally stressed mothers, the level of primary LP products decreased and the level of Schiff bases increased in the estrus stage. In the hippocampus of F2 females in the groups with prenatally stressed fathers, the level of Schiff bases decreased in the estrus stage, and the level of primary LP products increased in group 2 and decreased in group 4. In the hypothalamus of F2 females in the groups with prenatally stressed mothers, the level of Schiff bases increased in the estrus stage and decreased in the diestrus; in addition, in group 3, the level of primary LP products in the estrus stage increased. Thus, we demonstrated the influence of prenatal stress of both F1 mother and F1 father on the behavior and the level of LP in the neocortex, hippocampus and hypothalamus in female rats of the F2 generation in estrus and diestrus.

## Abstract (ru)

Исследовано влияние стресса у беременных самок крыс Вистар на поведение и показатели перекисного окисления липидов (ПОЛ) в неокортексе, гиппокампе и гипоталамусе у поколения самок F2 в течение эстрального цикла. Беременных самок подвергали ежедневному 1-часовому иммобилизационному стрессу с 15-го по 19-й день беременности. Далее из рожденных пренатально стрессированных и контрольных самцов и самок крыс поколения F1 формировали семейные группы: группа 1 – контрольные самка и самец, группа 2 – контрольная самка и пренатально стрессированный самец, группа 3 – пренатально стрессированная самка и контрольный самец, группа 4 – пренатально стрессированные самка и самец. Рожденных от этих семейных пар самок поколения F2 отбирали в четыре экспериментальные группы в соответствии с семейной группой. В возрасте трех месяцев у крыс исследовали показатели поведения в тесте «открытое поле» в двух стадиях полового цикла – эструсе и диэструсе. Через 7–10 дней крыс декапитировали и производили отбор неокортекса, гипоталамуса и гиппокампа для определения уровня диеновых и триеновых конъюгатов, оснований Шиффа и степени окисленности липидов (индекса Клейна). У самок F2 с одним пренатально стрессированным родителем отсутствует межстадиальная разница в локомоторно-исследовательской активности и тревожности. Если оба родителя F1 являются пренатально стрессированными, самки крыс F2 сохраняют межстадиальные различия, схожие с контрольным паттерном, при этом по абсолютным значениям у них снижаются локомоторно-исследовательская активность и время нахождения в центре открытого поля. В неокортексе у самок F2 в группах с пренатально стрессированными матерями снижается уровень первичных продуктов ПОЛ и повышается уровень оснований Шиффа в стадии эструса. В гиппокампе у самок F2 в группах с пренатально стрессированными отцами снижается уровень оснований Шиффа в стадии эструса, а уровень первичных продуктов ПОЛ повышается в группе 2 и снижается в группе 4. В гипоталамусе у самок F2 в группах с пренатально стрессированными матерями уровень оснований Шиффа повышается в стадии эструса и снижается в диэструсе, кроме того, в группе 3 повышается уровень первичных продуктов ПОЛ в стадии эструса. Таким образом, выявлено влияние пренатального стресса как матери F1, так и отца F1 на показатели поведения и уровень ПОЛ в неокортексе, гиппокампе и гипоталамусе у самок крыс поколения F2 в эструсе и диэструсе.

## Introduction

It has now been established that an increase in the level of maternal glucocorticoids during pregnancy causes changes in the neuroendocrine and immune systems of the offspring. Elevated level of maternal glucocorticoids promotes excess production of reactive oxygen species (ROS), with the organspecific stress response depending on the relative balance between ROS generation and the antioxidant capacity of the cell (Dennery, 2010; Thompson, Al-Hasan, 2012). Disruption of this balance leads to oxidative stress and contributes to epigenetic changes in prenatally stressed offspring. Epigenetic changes are maintained in a number of mitotic divisions of somatic cells, and can also be transmitted to the next generations if these changes occurred in germ cells (Dyban, 1988; Rodgers, Bale, 2015; Yao et al., 2021). Thus, numerous negative effects of maternal stress identified in the first generation may be sustained in subsequent generations (Essex et al., 2013; Provençal, Binder, 2015).

In females, both epigenetic changes and maternal behavior influence offspring, so studying the transgenerational effects of stress in male rodents has advantages compared to females (Brunton, 2013; Bale, 2014). In this regard, transgenerational changes caused by stress in females remain insufficiently studied. Currently, researchers are paying special attention to alteration of fertility in F2 and subsequent generations. Thus, a number of authors (Zhang et al., 2020; Piquer et al., 2022) found experimentally that prenatal nonphysiological influences of varying genesis affect fertility in both the second and third generations. This is expressed as a change in a number of morphometric parameters of the ovaries and uterus, biochemical parameters such as the level of corticosterone, luteinizing hormone, follicle-stimulating hormone, insulin and other metabolic parameters in the blood serum, as well as in disruption of the estrous cycle. Other studies have found that prenatal stress has a transgenerational effect on the processes of free radical oxidation of biomolecules in various tissues (Aiken et al., 2019).

changes have the potential to influence endocrine programming and brain development in the fetus over multiple generations. The authors of the review (Babenko et al., 2015) emphasize the complex relationship between the effects of prenatal stress on changes in microRNA expression, DNA methylation in the placenta and brain and an increased risk of developing mental illness

It can be assumed that prenatal stress, which causes epigenetic changes, becomes one of the most potent factors affecting mental health. Moreover, such changes affect, among other things, various structures of the brain associated with the neuroendocrine system and cognitive abilities. In the research (Huerta-Cervantes et al., 2021), it is noted that cognitive impairment in female rats at different stages of the ovarian cycle may be associated with disorders in the processes of lipid peroxidation in the hippocampus and neocortex.

Lipid peroxidation is not only a universal modifier of the properties of biological membranes, but also an important physiological regulator of their structure, a factor that establishes and supports the activity of enzymes, channel-former molecules, and receptors. The intensity of free radical processes of lipid peroxidation, which are under the control of endogenous oxidants, is associated with the composition and physical state of phospholipids of biological membranes (their fluidity), with their sensitivity to ligand signals and extreme influences. It is also extremely important for the regulatory and informational properties of membranes in normal cellular metabolism. Oxidative processes that affect the composition and viscosity of the lipid layer of membranes can regulate cellular metabolism (Baraboy et al., 1992).

Behavioral changes at different stages of the ovarian cycle in rats are associated with preparation for successful reproductive function. The changes in fertility may influence the behavioral features of different stages of the ovarian cycle. Thus, it is of interest to study the transgenerational effect of prenatal stress on behavior and lipid peroxidation in the brain structures of female rats during the ovarian cycle.

## Materials and methods

**Fig. 1.**
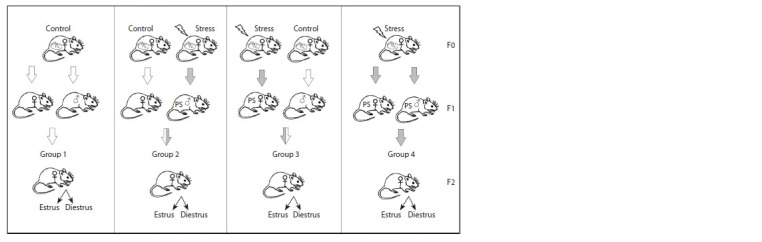
Experimental design. Group 1 – F2 offspring born from unexposed parents; group 2 – F2 offspring born from a mother not exposed to any influences and a prenatally stressed (PS) father; group 3 – F2 offspring born from a prenatally stressed mother and a father not exposed to any influences; group 4 – F2 offspring born from prenatally stressed parents.

For this study, the Wistar rats from the I.P. Pavlov Institute of Physiology of the Russian Academy of Sciences (St. Petersburg) were used. The recommendations on the ethics of working with animals proposed by Directive 2010/63/EU of the European Parliament and of the Council on the protection of animals used for scientific purposes were respected. The breeding protocol is presented in Figure 1.

Breeding of experimental groups of F2 offspring. Stage 1. The Wistar rats of the F0 generation weighing 280–300 g and three months old were used at the beginning of this stage. Rats were housed in standard cages for laboratory mice and rats M-6 (“Proflab”, Russia) and received rat water and chow for laboratory animals LBK-120 (Tosno, Russia) ad libitum. The animals were kept at a controlled temperature (22–24 ℃). A 12:12 h light-dark cycle was maintained. Female rats were coupled with males; fertilization was confirmed by the detection of spermatozoa in a vaginal swab and indicated as day zero of pregnancy. Pregnant females were randomly divided into two groups: control pregnant rats to breed control F1 offspring (n = 12) and rats that were further stressed to breed prenatally stressed F1 offspring (n = 12).

Stage 2. To breed prenatally stressed F1 offspring, pregnant F0 females were exposed to one-hour immobilization stress under high-light conditions from the 15th to the 19th day of gestation (Ordyan, Pivina, 2003). The stress was performed at the same time of day from 14.00 to 15.00 h. The days for stressing were chosen due to the fact that it is during this period that the integration of all parts of neuroendocrine regulation takes place and the formation of the hypothalamic-pituitaryadrenocortical system is completed (Rice, Barone, 2000).

Control and stressed pregnant females were housed 4–5 individuals in a cage. At the 20th day of pregnancy, the dams were housed in individual cages. The resulting offspring were counted on the 2nd day of life, taking into account the number of males and females in litters, and the litters themselves were aligned to 8–10 pups with an equal sex ratio. The pups were housed with their mother for 30 days and received water and chow ad libitum. Further, prenatally stressed F1 offspring were placed in cages, separating males and females. The control F1 offspring, born to intact F0 dames, were also aligned on the 2nd day of life and separated from the mother at the age of 30 days – males and females separately.

Stage 3. For breeding F2 offspring, animals were randomly selected by an independent person. The family groups of F1 offspring were formed from one male and three females, so that the animals were not siblings. These family groups were formed in such a way that the F2 offspring resulted in four experimental groups: group 1 (k+k) – offspring obtained from F1 females and F1 males from the control group that were not exposed to any influences, group 2 (k+2) – offspring obtained from F1 females of the control group and F1 males of the prenatally stressed group, group 3 (2+k) – offspring obtained from F1 females of the prenatally stressed group and F1 males from the control group, group 4 (2+2) – offspring obtained from F1 females and F1 males from the prenatally stressed group.

The resulting F2 offspring were counted on the 2nd day of life, taking into account the number of males and females in litters, and the litters themselves, as in the case of F1 offspring, were aligned to 8–10 pups with an equal sex ratio. The pups were housed with their mother for 30 days and received water and chow ad libitum. Next, the F2 offspring were placed in cages of 5–7 individuals, separating males and females. Females aged 3 months were used for further studies.

Previously, the rats were subjected to handling and trained: vaginal swabs were taken from them for 3 weeks. Next, the animals’ behavior was tested; immediately after testing, vaginal swabs were taken from rats and the stage of the ovarian cycle was determined. Two weeks after behavior testing, the rats were decapitated. Immediately after decapitation, control vaginal swabs were taken again.

Behavior testing. Open field. The “open field” test (OF) was a rectangular Plexiglas box (90 × 90 × 50 cm), the floor of which was divided into squares (15 × 15 cm). The illumination of the box was 300 lx. Testing occurred for 5 min from 10.00 to 13.00 h. The rat was placed in the center of the box and the total time in the center, the number of crossed squares (horizontal motor activity or locomotor activity), the number of vertical positions (vertical motor activity or research activity), the time of the grooming reaction and the time of immobility (freezing) were recorded. Indicators of horizontal and vertical motor activity indicate locomotor research activity. The total time in the center, the time of immobility and the reaction time of grooming indicate the degree of anxiety in rats

Determination of lipid peroxidation products. Rats were decapitated and the neocortex, hippocampus and hypothalamus were isolated on the ice. Next, lipids were extracted from the samples using the Folch method.

To determine the level of conjugated diene (CD) and triene (CT), the Klein index the dry lipid extract was dissolved in a methanol : heptane (2:1) mixture and the optical density was measured – the CD level at 230 nm and the CT level at 274 nm. The content of conjugated dienes and trienes was expressed in units of optical density per 1 mg of phospholipids (Arutyunyan et al., 2000). The fluorescent intensity of Schiff bases was determined by the fluorimetric method at a maximum excitation of 365 nm and a maximum emission of 425 nm (Bidlack, Tappel, 1973), expressed in relative units of fluorescence per 1 mg of phospholipids.

The amount of phospholipids was estimated by the content of nonorganic phosphorus by the Bartlett method. The method is based on the reaction of nonorganic phosphate with ammonium molybdate, resulting in phosphoric-molybdenum acid, which is then reduced by eikonogen to form colored molybdenum oxides, the optical density of which is measured at 830 nm.

To determine the degree of lipid oxidation, the Klein index was calculated as follows: the optical density of lipid extracts was determined at 215 nm and the ratio of optical densities at 233 and 215 nm was calculated.

To determine the level of conjugated diene and triene, the phospholipids and the Klein index, a BioTek PowerWave HT spectrophotometer (USA) was used. The determination of the Schiff bases level was carried out using a Hitachi MPF-4 spectrofluorimeter (Japan).

All reagents used in biochemical analyses were purchased from “Vecton” (Russia), with the exception of eikonogen (Merck, Germany).

Statistical analysis. For statistical analysis, STATISTICA 8.0 software package (StatSoft Inc.) was used. The normality of the distribution of values in the samples was determined using the Shapiro–Wilk test. Normally distributed data were analyzed with a parametric Student’s t-test and non-normally distributed data were analyzed with a non-parametric Mann– Whitney U-test. Data that are presented as Mean ± SEM are indexes of behavior, and those presented as medians (Me) and interquartile range (IQR) between the values of 25 and 75 percentiles are biochemical indexes. To identify differences between ovarian cycle stages in each of the four studied groups, comparisons of each indicator in the estrus and diestrus stages were performed. And also the values of each index in groups 2, 3 and 4 were compared with group 1 both in estrus and in diestrus. The differences were considered statistically significant at p < 0.05.

## Results

Behavior

**Fig. 2.**
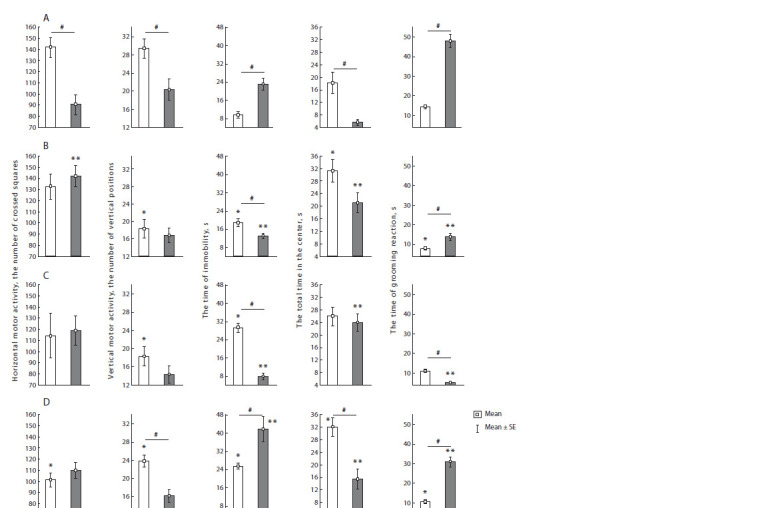
Rats behavior in the “open field” test. Light bars are rats in estrus, dark bars are rats in diestrus. Panel A – group 1 (control rats); panel B – group 2 (control mother and PS father); panel C – group 3 (PS mother and control father); panel D – (PS parents). # – statistically significant differences between rats in diestrus and estrus, p < 0.05; * – statistically significant differences from the control in animals in estrus, p < 0.05; ** – statistically significant differences from the control in animals in diestrus, p < 0.05.

Differences in behavioral indicators between ovarian cycle stages are observed in the group of control animals (Fig. 2).

The indicators of horizontal motor activity, vertical motor activity and time of presence in the center of OF in females in estrus are higher than in females in diestrus, while the time of immobility and grooming time are lower in females in estrus. It can be concluded that in the control group, females in estrus have increased motor and research activity and decreased anxiety (according to the indicators in the OF test), which, apparently, represents an evolutionarily appropriate strategy related to sexual behavior

In the k+2 group, horizontal and vertical motor activities, as well as time of presence in the center in female F2 rats, do not differ in estrus and diestrus. The indicators “freezing” and “grooming reaction time” demonstrate an interstadial difference. F2 females in diestrus become more active and less anxious compared to the control. In F2 females, research activity decreases in estrus, the freezing time increases, the time present in the center increases; that is, the females become less anxious. Based on the changes described above, we assume that F2 females, during the most favorable period for mating, become less mobile compared to the control and, accordingly, the likelihood of meeting a partner decreases. At the same time, females in diestrus, according to the studied behavior indicators, approach females in the estrus stage in the control group

In group 2+k, the indicators of horizontal and vertical motor activity and time in the center in female F2 rats do not differ in estrus and diestrus. The indicators “freezing” and “grooming reaction time” demonstrate the difference between estrus and diestrus, which is inverted with respect to the control. F2 females in diestrus are less active, spend less time on grooming and prefer to be in the center of the OF, i. e. they have reduced anxiety compared to the control. Research activity decreases and the time of immobility increases in F2 females in estrus compared to the control; in addition, females of this group are less mobile in estrus than in diestrus. Thus, in this group, too, females in estrus have a distortion of the behavior associated with finding a partner

In the 2+2 group, female F2 rats have shown differences between the stages of the ovarian cycle in all indicators except horizontal motor activity. F2 females in diestrus are less mobile and more anxious compared to the controls. In F2 females in estrus, there is also a decrease in locomotor and research activity and an increase in anxiety compared to the control. Nevertheless, the interstadial ratio in females of this group remains similar to the interstadial ratio in the control group; however, according to the absolute values of behavior indicators, females of the 2+2 group differ from the control group by an increase in anxiety

Neocortex

**Fig. 3.**
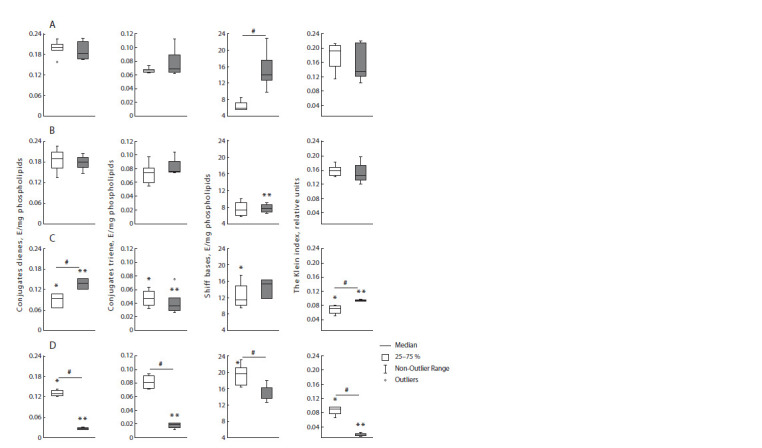
CD, CT, Schiff bases levels and Klein index in the neocortex of female rats in the experimental groups Light bars are rats in estrus, dark bars are rats in diestrus. Panel A – group 1 (control rats); panel B – group 2 (control mother and PS father); panel C – group 3 (PS mother and control father); panel D – (PS parents). # – statistically significant differences between rats in diestrus and estrus, p < 0.05; * – statistically significant differences from the control in animals in estrus, p < 0.05; ** – statistically significant differences from the control in animals in diestrus, p < 0.05.

In the control group, there are differences between the stages of the cycle only in Schiff bases: in the estrus stage, the level of the end products of lipid peroxidation reactions is 2 times lower than in the diestrus stage (Fig. 3).

In the k+2 group, the Schiff bases level in diestrus is two times smaller than in the control group; however, the indicators of other studied products of lipid peroxidation do not differ from the level of the control group. There are no interstadial differences.

In the 2+k group, in F2 females, relative to the control indicators, the level of CD and CT – the initial products of lipid peroxidation – is lower, and the level of Schiff bases (the final products) is higher. It should be noted that the CD indicators and the Klein index show an interstadial difference

The levels of CD, CT and the Klein index in the 2+2 group are lower relative to the corresponding control indicators in both stages of the cycle, with the exception of CT in estrus. The Schiff bases level in estrus is 3 times higher than in the control group. It can be concluded that in the 2+2 group, the level of lipid peroxidation indicators is lower compared to the control, especially in the diestrus stage, excluding the Schiff bases. It should be noted that all the studied lipid peroxidation indicators of this group demonstrate a difference between estrus and diestrus.

Thus, the k+k and k+2 groups are similar in their profile of levels of CD, CT and the Klein index in the neocortex, whereas groups 2+2 and 2+k differ by a decrease in these indicators of lipid peroxidation. At the same time, the Schiff bases indicators of these groups in the estrus stage are significantly higher than the control indicators, whereas in diestrus, only the k+2 group is characterized by an acute decrease in this indicator

Hippocampus

**Fig. 4.**
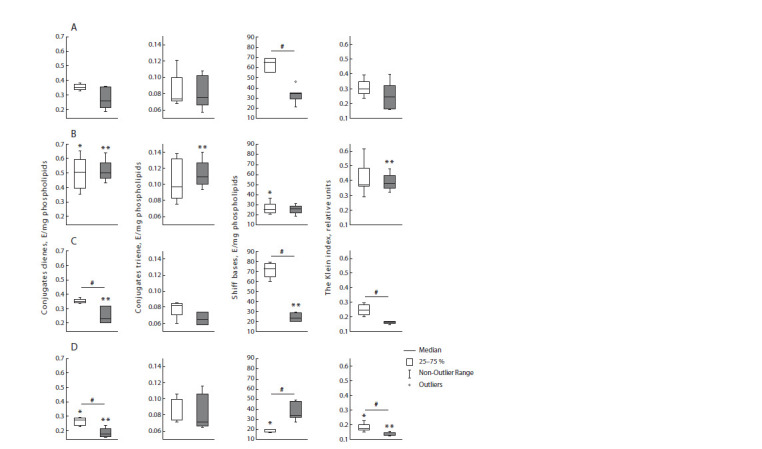
CD, CT, Schiff bases levels and Klein index in the hippocampus of female rats in the experimental groups. Light bars are rats in the estrus stage, dark bars are rats in the diestrus stage. Panel A – group 1 (control rats); panel B – group 2 (control mother and PS father); panel C – group 3 (PS mother and control father); panel D – (PS parents). # – statistically significant differences between rats in diestrus and estrus, p < 0.05; * – statistically significant differences from the control in animals in estrus, p < 0.05; ** – statistically significant differences from the control in animals in diestrus, p < 0.05.

In the control group, there are differences between the stages of the cycle only in the Schiff bases level: in the estrus stage, the level of the end products of lipid peroxidation is two times higher than in the diestrus stage (Fig. 4).

In the k+2 group, the CD level in estrus and diestrus is higher than in the control group, the CT level in diestrus is higher than in the control group. The Schiff bases level in estrus is two times lower than in the control group. The values of the Klein index in diestrus are higher than in the control. It can be concluded that the level of indicators of the initial lipid peroxidation products is higher compared to the control group, especially in diestrus, but this group is characterized by the absence of differences between estrus and diestrus.

In the 2+k group, the Schiff bases level in the diestrus stage is lower relative to the control group. In diestrus, there are no differences between CD, CT and the Klein index relative to the control indicators. At the same time, there is a difference between estrus and diestrus for Schiff bases, CD, and the Klein index

The CD levels in the 2+2 group in both stages of the ovarian cycle are lower relative to the values in the control group. The CT level is characterized by the absence of both differences between the ovarian cycle stages and differences from the control group. The Schiff bases level in estrus is 3 times lower compared to the control group, and there is no difference in diestrus compared to the control. The Klein index in estrus and diestrus is lower relative to the respective control indicators. In group 2+2, the level of lipid peroxidation indicators is lower compared to the control and there are differences between estrus and diestrus in the levels of both initial and final lipid peroxidation products. It is noteworthy that the 2+k and 2+2 groups demonstrate a difference between estrus and diestrus in lipid peroxidation indicators.

Hypothalamus

**Fig. 5.**
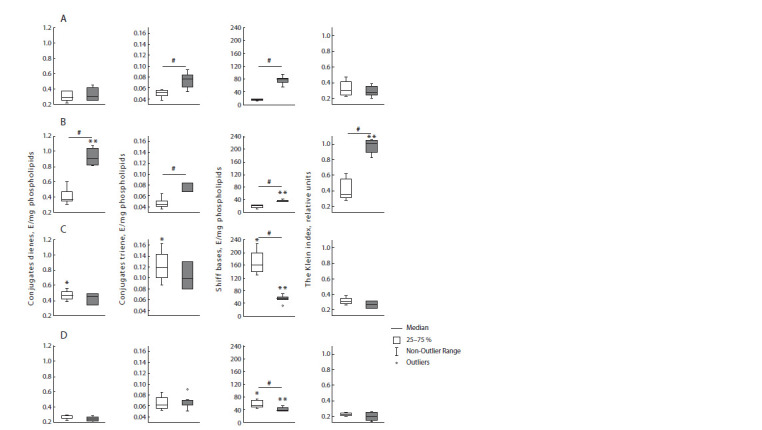
CD, CT, Schiff bases levels and Klein index in the hypothalamus in female rats in the experimental groups. Light bars are rats in estrus, dark bars are rats in diestrus. Panel A – group 1 (control rats), panel B – group 2 (control mother and PS father), panel C – group 3 (PS mother and control father), panel D – (PS parents). # – statistically significant differences between rats in diestrus and rats in estrus, p < 0.05; * – statistically significant differences from the control in animals in estrus, p <0.05; ** – statistically significant differences from the control in animals in diestrus, p < 0.05.

In the control group, the levels of CT and Schiff bases differ between estrus and diestrus. The level of these lipid peroxidation products is higher in diestrus compared to estrus (Fig. 5).

In the k+2 group, the Schiff bases level in diestrus is lower than in the control group. The CD level and the Klein index in diestrus are 3 times higher than the control level, and the CT level does not differ. Interstadial differences are observed for all the studied lipid peroxidation indicators in k+2 group.

In the 2+k group, the Schiff bases level in estrus is 10 times higher relative to the control level, but in diestrus it is lower. The levels of CD and CT in estrus are higher compared to the control group. The Schiff bases level has a difference between estrus and diestrus inverted with respect to the control.

The Schiff bases level in the 2+2 group in estrus is 3 times higher than in the control group, whereas in diestrus, the Schiff bases level is 2 times lower than in the control. There are no differences in other lipid peroxidation indicators between estrus and diestrus. Also there are no differences in these indicators of the control group.

The similarity of the k+k and 2+2 groups in the level of initial and intermediate lipid peroxidation indicators is noteworthy. At the same time, the Schiff bases levels of the 2+2 group in estrus and diestrus are inverted relative to the control. Also noteworthy is the multiple increase in comparison with the control of the initial lipid peroxidation products and the oxidation index (Klein index) in diestrus in the k+2 group, and the Schiff bases level in estrus in the 2+k group.

## Discussion

An increase in maternal glucocorticoid levels during pregnancy can lead to sustainable epigenetic changes. In the research (Gilbert et al., 2012; Matthews, Phillips, 2012), it is noted that epigenetic changes may be sustained over subsequent generations

Due to the changed social status of women in recent decades, there has been a shift in the reproductive age to later age cohorts. The presence of a significant number of assisted reproductive technologies (ART) allows women to procreate even in the case of significant pathologies. However, the negative consequences of such pathological pregnancies for future generations are currently only beginning to be understood (Aiken et al, 2015; Sanches-Garrido et al., 2022).

In the review by A.L. Levinson et al. (2022), the authors note the multifactorial nature of hormonal effects in the reproductive dysfunction problem. When analyzing the failures of the use of ART, the importance of not only hypothalamic disorders, but also the influence of paracrine factors of the ovary has been revealed. At the same time, in our laboratory’s study, it was found that the negative effect of prenatal stress on the morphometric parameters of the uterus associated with a disturbance of the cycle of sex hormones is noted only in young female rats aged 3 months, while in older animals such disruptions are leveled (Pivina et al., 2010). However, in a study by B. Piquer et al. (2022), a decrease in the fertility of female rats has been noted, expressed in impaired fertilization and the number of pups born after prenatal stress up to the F4 generation. The same authors have shown a disturbance of the morphometric parameters of the ovaries and uterus and disorders of the ovarian cycle before the F4 generation. As A.L. Levinson et al. (2022) note, “Despite the fact that research on the effects of psycho-emotional stress is widely represented in both the medical and scientific biological literature devoted to experiments on laboratory models, these two areas are developing largely independently”. It can be suggested that failures in the use of ART may be caused, among other things, by the transgenerational effects of stress.

In various experimental models of transgenerational transfer of epigenetic changes, fertility disorders of female offspring over several generations are noted (Guilbert et al., 2012; Moisiadis et al., 2017; Adams, Smith, 2020). In addition, the effect of prenatal stress on transgenerational changes in male and female offspring is different (Grundwald, Brunton, 2015; Zaidan, Gaisler-Salomon, 2015; Zhang et al., 2020; Huerta- Cervantes et al., 2021). At the same time, both prenatally stressed mothers and fathers have an impact

According to our data, the behavior of control group 1 females in diestrus is characterized by increased anxiety indicators, whereas in estrus anxiety is reduced and locomotor and research activity is increased. These data correspond to earlier studies (Mora et al., 1996; Marcondes et al., 2001; Miller et al., 2021), where behavioral changes in estrus and diestrus are associated with a different hormonal profile. Obviously, the relationship is the result of the fact that receptors for sex hormones are present in the structures of the brain, causing evolutionarily appropriate behavioral reactions associated with reproduction (Reznikov et al., 2004).

A change in the lipid peroxidation level is considered an important indicator of membrane destabilization (Levitsky, Gubsky, 1994) and can cause an alteration of the molecular structure of membranes, which in turn is expressed as a change in behavior (Moisiadis et al., 2017). At the same time, lipid peroxidation processes, occurring within the physiological norm, represent a mechanism for regulating the physicochemical state of membranes and, accordingly, structures associated with membranes – receptors and ion channels (Halliwell, Gutteridge, 2007). The data obtained in our study allow us to conclude (based on changes in the Schiff bases level) that in estrus there is a decrease in viscosity and an increase in plasticity of membranes in the neocortex and the reverse changes occur in the hippocampus in rats. In the hypothalamus in estrus, changes in the level of lipid peroxidation, and, accordingly, changes in the physicochemical state of the membranes are similar to those in the neocortex, but more pronounced. Apparently, changes in behavior in estrus compared to diestrus require appropriate changes in membranes and related structures (receptors and ion channels).

When interpreting the results, the model of “father prenatal stress” is more understandable for studying the mechanisms of epigenetic transfer than “mother prenatal stress”, which has additional effects on F2 offspring by maternal behavior, childbirth and lactation (Bale, 2015). The detection of an altered phenotype in the case of an experiment with a prenatally stressed father can be considered valid evidence of transgenerational transfer of epigenetic changes in the second generation (Dunn et al., 2011).

Our study showed that in group 2, where one of the parents is a prenatally stressed father, behavior at different stages of the ovarian cycle does not correspond to the goal of reproductive behavior, accordingly, we can make an indirect conclusion about a disturbance of the hormonal regulation of sexual behavior. Considering the results of the lipid peroxidation change, we see that the physico-chemical properties of the neocortex membranes in rats in diestrus are characterized by an increased level of membrane plasticity in terms of the Schiff bases level in comparison with the control. In the hippocampus, changes in different lipid peroxidation products are multidirectional compared with the control, but taking into account such an indicator as the Klein index, which characterizes the degree of lipid oxidation, it can be concluded that the level of lipid peroxidation in diestrus increases compared with the control group. At the same time, in the hypothalamus, changes in lipid peroxidation indicators in estrus compared with diestrus are similar to the control, but more expressed.

Thus, changes in the level of lipid peroxidation in the studied brain structures probably also contribute to changes in the sexual behavior of females in a group where one of the parents is a prenatally stressed father. It can be concluded that the father’s prenatal stress makes an important contribution to the reproductive pattern of the daughters, including through biochemical processes associated with the oxidation of biomolecules

Maternal prenatal stress is an additional stressor because F2 offspring are cared for by a female with impaired maternal behavior (Graf et al., 2012). However, the behavior of group 3, where the mother was subjected to prenatal stress, demonstrates a distortion of the behavior of females in both estrus and diestrus, similar to group 2. Lipid peroxidation processes in the neocortex demonstrate an imbalance between the initial and final products, due to which changes occur compared to the control. Thus, a difference between estrus and diestrus appears in the indicators of conjugated dienes and the Klein index when these indicators decrease relative to the control ones. While the indicator of the final products of the lipid peroxidation – Schiff bases – in the estrus stage exceeds the values in the control group, as a result of which the interstage difference disappears.

In the hippocampus, the main changes relate to a decrease in the lipid peroxidation indices in diestrus relative to the control, whereas in the hypothalamus, on the contrary, the lipid peroxidation indices increase in estrus relative to the control values. Apparently, behavioral disorders at different stages of the ovarian cycle may occur in this group, including as a result of changes in the physico-chemical properties of the membranes of the researched structures: an imbalance in the neocortex and interstage distortions of the lipid peroxidation processes in the hippocampus and hypothalamus (an increase in the plasticity of the hippocampal membranes in diestrus and a decrease in the plasticity of the hypothalamus membranes in estrus). Thus, a prenatally stressed mother affects changes in the reproductive pattern of daughters differently at different stages of the cycle. Likely, possible epigenetic changes in F2 females are also influenced by disturbances in maternal behavior in prenatally stressed F1 females

There is information in the literature on the cumulative effect of prenatal stress of both parents on offspring (Adams, Smith, 2020). In our studies in group 4, where both parents were exposed to prenatal stress, the behavior of female F2 offspring shows an increase in anxiety in both estrus and diestrus. Lipid peroxidation in the neocortex of this group undergoes changes compared to the control group, due to which there is a significant interstadial difference in all indicators of lipid peroxidation. A similar profile of interstadial differences is observed in the hippocampus. In the hypothalamus, Schiff bases levels are inverted by stage relative to the control group. It can be assumed that one of the reasons for the increase in anxiety, regardless of the stage of the ovarian cycle in this group, may be a change in lipid peroxidation processes in the neocortex and hippocampus. Perhaps the cumulative effect of prenatal stress of both parents is manifested in this group by an unambiguous change in behavior at both stages of the cycle and impairment of lipid peroxidation in the neocortex.

## Conclusion

Our results revealed the transgenerational effect of prenatal stress on the processes of lipid peroxidation and the behavior of female rats of the F2 generation, depending on the stage of the ovarian cycle.

The prenatal stress of the father or mother changes the processes of lipid peroxidation in the neocortex, hippocampus and hypothalamus of a female rat of the F2 generation in such a way that physico-chemical properties of the membranes of these brain structures do not correspond to the goals of the ovarian cycle stages. While the prenatal stress of the father causes the greatest changes in the hypothalamus lipid peroxidation processes, and the prenatal stress of the mother – those in the neocortex. The behavior in both cases does not meet the objectives of reproduction.

Prenatal stress of both parents of the female rats of the F2 generation has the greatest effect on the changes in lipid peroxidation processes in the studied brain structures, reducing the intensity of lipid peroxidation. The behavior is characterized by increased anxiety in both the researched ovarian cycle stages.

## Conflict of interest

The authors declare no conflict of interest.
